# Inferring Opinions and Behavioral Characteristics of Gay Men with Large Scale Multilingual Text from Blued

**DOI:** 10.3390/ijerph16193597

**Published:** 2019-09-26

**Authors:** Ge Huang, Mengsi Cai, Xin Lu

**Affiliations:** 1 College of Systems Engineering, National University of Defense Technology, Changsha 410073, China; gehuang_nudt@hotmail.com (G.H.); caimengsi18@nudt.edu.cn (M.C.); 2 School of Software Engineering, Shenzhen Institute of Information Technology, Shenzhen 518172, China; 3 School of Business, Central South University, Changsha 410083, China

**Keywords:** gay men, content analysis, behavioral characteristics, GSN apps, Blued

## Abstract

Gay men in many countries are increasingly using geosocial networking applications (GSN apps), thus offering new opportunities for understanding them. This paper provides a comprehensive content analysis of posts and opinions on Blued, the world’s largest gay social networking dating app, to infer and compare opinions and behavioral characteristics of gay men in different countries. Machine learning and linguistic programming approaches were used to extract themes and analyze sentiments of posts. The results show that the majority of posts are related to daily life activities, and less are related to sensitive topics. While most posts are positive or neutral, negative emotions, including anxiety, anger, and sadness, are mainly distributed in posts related to self-identification and sexual behaviors in China and to relationships in other countries. Voting items indicate that only 50.52% of the participants will take regular HIV tests while 50.2% would have casual sex when they are single. Additionally, 35.8% of the participants may try drugs when invited by friends. Our findings suggest an opportunity and necessity for researchers and public health practitioners to use open source data on GSN apps and other social medias to inform HIV interventions and to promote social inclusion for sexual minorities.

## 1. Introduction

Social media provide an important platform for people to communicate and interact online. As new technologies are developed, geosocial networking applications (GSN apps) on mobile phones are gradually replacing traditional social media in people’s lives. GSN apps are diverse with regard to their customer bases, points of interest, and operator interfaces. Studying the usage patterns of social media or applications for specific populations allows us to better understand their behavior and inform interventions [1,2]. In sexual minorities, GSN apps, such as Blued, Grindr, and HER, have been commonly used since 2009, helping individuals from these groups to socialize, seek partners, and obtain support. For example, the world’s largest GSN APP, Blued, originating from China, is now a very popular social media worldwide, especially in the Asia-Pacific region. However, recent data from The Joint United Nations Programme on HIV/AIDS (UNAIDS) show that, since 2016, the rate of new human immunodeficiency virus (HIV) infections in the Asia-Pacific region has stopped decreasing. According to the analysis, about 30% of new infections in the Asia-Pacific region occur in men who have sex with men (MSM). HIV prevalence and infection rates have risen sharply among young men who have sex with men (MSM) in countries, such as China and Indonesia [3].

GSN apps provide an effective social platform for sexual minorities, as well as a potential tool for studying their high-risk behaviors [4]. Recent research has examined the sociodemographic characteristics [5], app usage [6], sexual behaviors [7], HIV testing and detection [8,9], drug and alcohol use [10], and efficiency of recruiting MSM using apps [11,12]. It is easily understood why researchers link app usage to high-risk behavior, but the viewpoints and emotions of gay men shown in the discourses have largely been ignored [13]. GSN apps contain a large amount of textual information, which plays a vital role in understanding the behavior and psychological conditions of their users. However, few researchers have paid attention to the text content generated on GSN apps [14,15]. Thus, in this paper, we utilized the textual content generated on a popular GSN app to infer opinions and behavioral characteristics of gay men.

To analyze the discourses generated on social networks, such as tweets and posts, two representations have been widely used. First, theme extraction is commonly employed to characterize the topics being discussed on social media [16,17,18]. The theme extraction methods are diverse due to the differences in the lengths of texts, the amount of data, and the granularity needed. Second, sentiment values and linguistic styles have been found to be helpful in understanding users’ viewpoints [1,19], which generally include lexicon-based and machine learning-based approaches. These two representations enable us to document what users are interested in and how they discuss their interests, providing insights into their life quality and mental status.

In this work, we used a large-scale multi-language text dataset on a popular GSN app for gay men named Blued, which included 1186 topics and all voting items. We focused on three major aspects: (1) What is the thematic content of the posts? (2) what is the sentiment of the posts in each category? and (3) what are the users’ views on sensitive issues? Statistical, machine learning, and linguistic programs were used in the analysis.

A key contribution of this paper is to provide a comprehensive view of a GSN app through topic classification as well as sentiment and linguistic analysis. Another contribution is to document the opinion and behavior characteristics of a large number of gay men who use GSN apps. This study demonstrates the potential of GSN apps as a new channel for gay behavior monitoring. Our findings provide data support for research and suggest the potential of machine learning classification and psychoanalysis techniques in the textual content analysis for sexual minorities.

## 2. Data and Methods

### 2.1. Data Collection and Filtering

Blued is a GSN app for gay men that allows users to post and interact through short messages. It was first created for Chinese gay men in 2012 but has since become the largest GSN app for gay men worldwide, with over 40 million users, especially in the Asia–Pacific region. According to the users’ locations, the Blued app is divided into Blued (only for gay men in mainland China) and Blued international (for gay men in other parts of China and other countries). The topic data in the Chinese language and all voting data were collected from Blued, while the topic data in non-Chinese languages were collected from Blued international from 1 January 2015 to 28 February 2019. 

**Figure 1:**
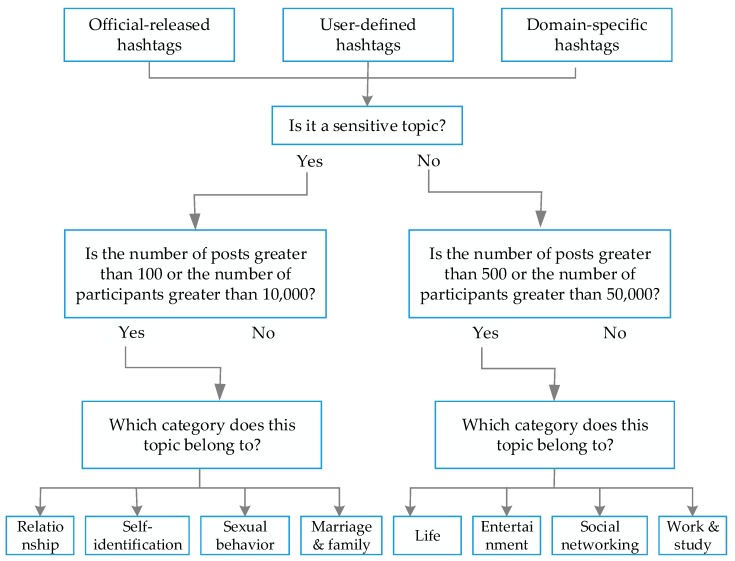
Flow of topic collection, filtering, and classification.

The topic data were collected by searching posts with specific hashtags in the “Topic List” module in Blued. A hashtag is a type of metadata tag that is widely used on many social networking sites and apps. With Blued, users can create and use hashtags in different languages by placing the pound sign (#) in front of a word or unspaced phrase in a non-Chinese post (e.g., #love) or by placing the pound sign both before and after words in a Chinese post (e.g., #爱自己#). Figure 1 shows the flow of topic collection, filtering, and classification used in this study. We divided hashtags into official-released, user-defined, and domain-specific hashtags. For official-released hashtags, we collected Chinese and English hashtags displayed in the “discovery” interface in Blued and hashtags released by official accounts. For user-defined hashtags, we first collected the 800 most commonly used words in Chinese. Then, we collected the 500 most popular hashtags on Instagram from a website that lists the most popular hashtags on various social networks (Top-Hashtags.com). For domain-specific hashtags, we collected sensitive topics by searching sensitive words related to gay men using the topic search interface in the app, based on our prior knowledge. Then, we used these words and unspaced phrases with the topic search interface on Blued and recorded the hashtags with a large amount of discussion or participation. After the hashtags were identified, we collected all the posts containing them. Non-English hashtags were not considered because trying to find keywords to use to search in 97 languages was not feasible. Up to March 2019, a total of 1186 topics were collected, including 551 Chinese topics and 635 English topics. 

For further screening, we divided the topics into sensitive topics-related to love, self-identification, high-risk behaviors, marriage, and family; and non-sensitive topics-related to life, entertainment, friendship, work, and study. Since most of the daily discussions of the users involved non-sensitive topics, we removed non-sensitive ones with less than 500 posts or with less than 50,000 participants (users’ behaviors include creating posts, upvoting, or replying to posts) as well as sensitive topics with less than 100 posts or with less than 10,000 participants for subsequent analysis. After removing the unimportant topics, we were left with 1132 topics, including 511 Chinese topics and 621 English topics. The Chinese topic data included 1,100,324 posts while the English topic data included 1,994,167 posts. It must be noted that the topic interface in Blued is a Twitter-style interface that most Chinese users used to release a post with or without a hashtag. While the topic interface in Blued international is an Instagram-style interface, most non-Chinese users tend to incorporate multiple hashtags in a post to increase the exposure of themselves, and then to make more friends. Consequently, there were many duplicate posts in the topic data, especially in non-Chinese posts. After we removed duplicates, 931,109 Chinese posts and 738,203 non-Chinese posts remained for further analysis.

Voting is an important function that reflects the common viewpoints and attitudes of people using social networks. It generally consists of a question with two or more options, and some user comments. “Viewpoint” is a sub-module in an earlier version of the Blued app (the direct entry has now been removed), and the voting questions posted in the module are usually related to sensitive topics. In this work, we collected all the voting data from the “Viewpoint” module in Blued, including 114 voting items, 5,042,240 votes, and 407,453 comments (see Table A1).

### 2.2. Classification

Three of the authors examined the topic dataset independently then discussed and summarized the classification framework. The final categories and selected topic data examples are listed in Table 1, in which all topics are classified into the following eight categories: Life, entertainment, social networking, relationship, self-identification, high-risk behavior, work and study, and marriage and family.

### 2.3. Sentiment and Psychological Analysis

**Figure 2:**
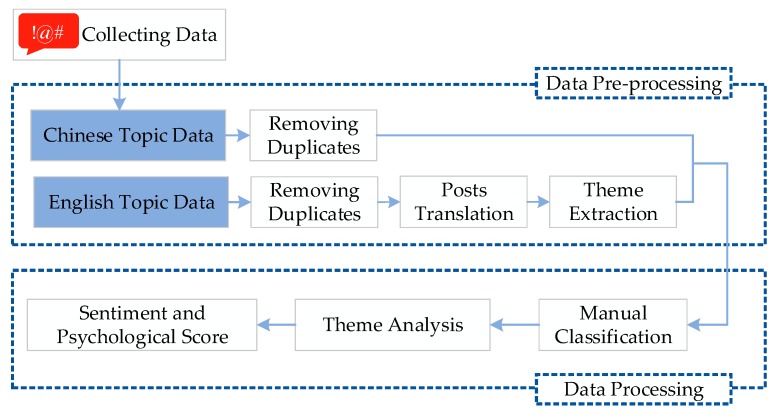
Architecture of the analysis process of the topic data.

As most Chinese posts included only one hashtag, we classified them manually based on the subject of the hashtag. For the English topic data, we translated all non-English posts to English and used a semi-automatic method to extract themes from the posts. Then, we used psycholinguistic programs to calculate the total number of words each theme used that fell into each affection category and classified the affections expressed in each post. Figure 2 shows the analysis process for the topic data. For voting items, we classified them and identified the main points from the text manually.

#### 2.3.1. Manual Classification of Chinese Topic Data

Due to differences between Chinese and other languages, the design and use of hashtags in the Chinese language are very different from the design and use of hashtags in other languages. Each Chinese hashtag has a specific meaning (e.g., #ideal lover#, #my future#, #mom, #I want to tell you) while most English hashtags have no specific meaning (e.g., #gay, #beach, #city). In addition, when publishing a post, Chinese users generally include only one topic hashtag, but most non-Chinese users tend to incorporate multiple hashtags in a post. As a result, the contents of posts in Chinese are more relevant to the hashtags that they are tagged with. Thus, we manually categorized all Chinese topic hashtags according to the classification frame.

Two of the authors independently examined 25% of the hashtags. If a single hashtag contained multiple themes, the hashtag was classified according to the predominant theme of the post to which it was attached. Next, the authors established a classification framework together to avoid ambiguities in category definitions. Finally, the two authors re-examined all the hashtags separately and resolved any differences on classification through discussion.

#### 2.3.2. Semi-Automatic Classification of English Topic Data

When we searched posts by English hashtags, the results contained a large number of posts written in a total of 97 different non-English languages, especially languages from Southeast Asia (see Table 2). Therefore, we developed a post-flow translation code program based on Google Translate and translated all non-English topic posts into English. As mentioned above, the hashtags attached to each non-Chinese post were numerous and had low relevance to the subject of the post, so it was not possible to categorize the posts according to the content of the hashtags directly. Therefore, we used machine-learning methods to cluster the non-Chinese posts.

We examined the non-Chinese posts and found that, despite having attached hashtags, most of the posts were short, and single posts tended to be about a single theme. Since the standard latent dirichlet allocation (LDA) method generally does not work well with short texts, we applied a modified author-topic model named Twitter-LDA [20], which assumes a single subject assignment for each post. We removed posts that only contained hashtags (more than three) and those from users with fewer than three posts. Then, we used the Twitter-LDA model to automatically extract 110 topic clusters (based on preliminary experiments) across 500 iterations from all the posts. After the noisy topics were removed, we obtained a set of 104 topic clusters, which we manually assigned to one of the eight topic categories mentioned above.

#### 2.3.3. Automatic Sentiment and Psychological Analysis

To measure the sentiment and psychological status of each topic category, we used a computational linguistics program called Linguistic Inquiry and Word Count (LIWC) [21] to analyze non-Chinese posts and a modified Chinese LIWC program called TextMind [22] to analyze Chinese posts. Both programs calculate the percentages of words in a given text that fall into one or more of over 80 linguistic, psychological, and topical categories indicating various cognitive, social, psychological, and affective processes. These programs, for example, help users to determine the degree to which a text uses pronoun or verb, or positive or negative emotions. In this work, we focused on categories related to affections. It must be noted that the core of the programs are dictionaries containing words that belong to predefined categories and the different LIWC indicators are not statistically independent of each other. For example, the word “afraid” is classified into *Anx* (represented for anxiety) category, the *Anx* is a subcategory of *NegEmo* (representing negative emotion), and the *Negative Emotion* is a subcategory of *Affect* (representing affection). The output features related to affective processes are as follows: *Affect, PosEmo, NegEmo, Anx, Anger*, and *Sad*. We counted and compared the output measures of the eight categories of topics separately for the Chinese and English topic data.

#### 2.3.4. Classification of Voting Data

We used the same method described in Section 2.3.1 to manually categorize all voting items according to our classification frame. As there were no voting items related to entertainment, we classified all voting items into seven categories. We examined the number of voting items and users involved, the number of comments in each category, and then summarized the main points of each category.

### 2.4. Ethics Approval and Consent to Participate

The study and Liu (2018) [13] were both supported by the Natural Science Foundation of China (91546203, 71771213) and approved by the Medical Ethical Committee of the Institutional Review Board (IRB) at Peking University (IRB00001052–16016). The study did not involve any physical, social, or legal risks to the participants; the data is anonymous; and the confidentiality of the participants’ information was strictly protected.

### 2.5. Data Availability

All data analyzed in this study are publicly available; all posts in the datasets can be collected by searching hashtags in the “topic” interface on Blued. Other data that support the findings in this study are available from the corresponding author on reasonable request.

## 3. Results

### 3.1. Topic Data Analysis

#### 3.1.1. Basic Statistical Results

Chinese topic data: There were 511 topics published in Chinese, involving 931,109 posts. There was no significant difference between the distribution characteristics of Chinese topics and those of Chinese posts. As illustrated in Table 3, more than half of the posts were related to daily life, such as publishing photos or videos. The second popular theme was relationships, accounting for 19.2% of the posts, followed by self-identification and entertainment topics, accounting for 7.7% and 6%, respectively. Social networking topics accounted for 3%, and the proportion of the other three topics was less than 2%. Because Blued is a GSN app for gay men, most users are keen to discuss topics closely related to life and love. Due to the particularity of their sexual identity, they also discuss self-identity and sexual behavior. However, only 9.1% of posts shared information about gay identity, and very few posts involved sexual behavior in gay men (<1%). On the one hand, the results revealed the users’ motives of “making friends” and “attracting attention”. They hoped to increase connection between each other by sharing information about daily lives, and then meet more gay friends. On the other hand, the users tended to be reserved in relating private information, such as high-risk behavior and HIV status, likely because disclosure of this information might have a negative impact on their socialization.

English topic data: After removing noisy topics, 104 English topics remained, involving 532,181 posts. As illustrated in Table 3, the distribution characteristics of English topics were not significantly different from those of the Chinese topics. The majority of non-Chinese posts were about life, accounting for 74.9% of the posts, followed by social networking and relationship topics, accounting for 7.4% and 7.2%, respectively. An additional 2% of posts reflected self-identification. A smaller number of posts reflected sexual behavior (1.4%) and work and study (1.1%) topics. Very few posts involved marriage and family (<1%). Interestingly, sensitive topics accounted for a lower proportion of total posts in non-Chinese posts than in Chinese posts. The results show that non-Chinese users are more likely to use the software for making friends, and their countries are more open to sexual identity, so these users are less likely to discuss sexual identity and sexual behaviors.

#### 3.1.2. Theme Analysis

**Figure 3:**
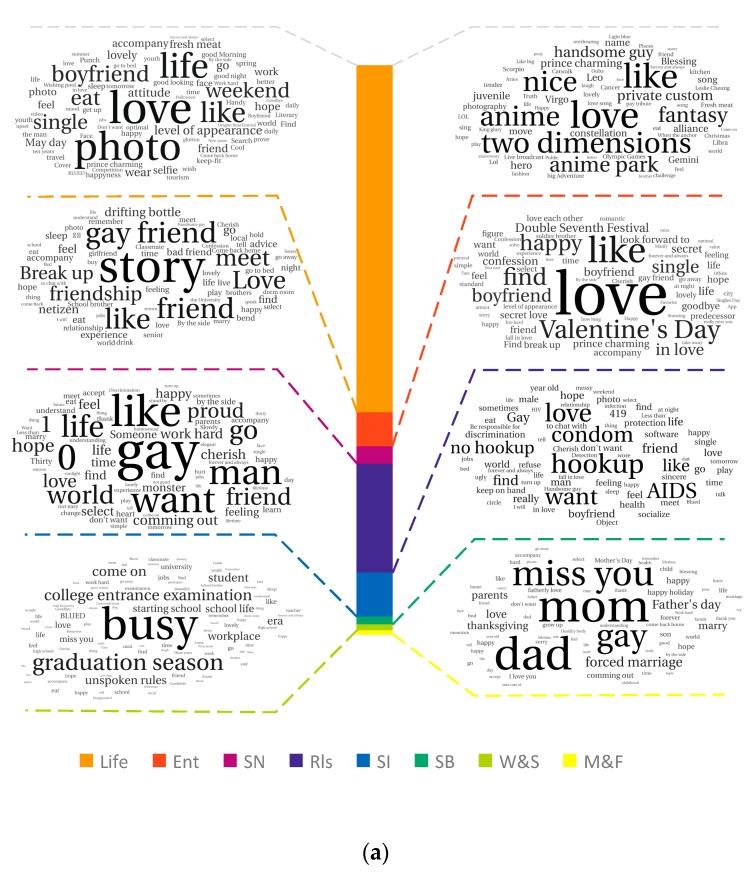

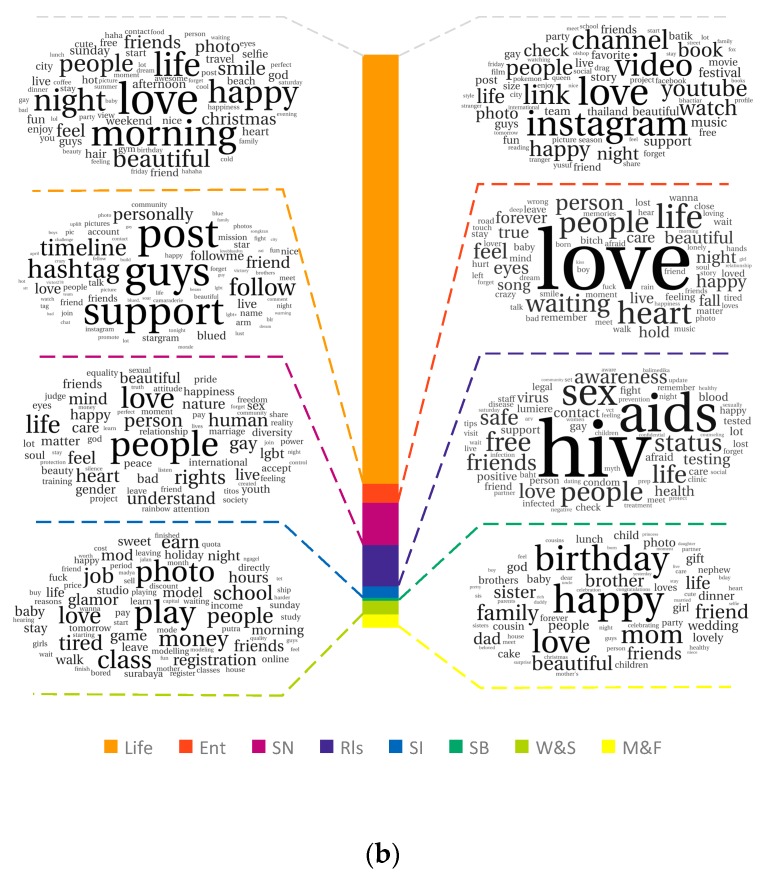
Word cloud of the eight categories of (**a**) Chinese and (**b**) English topic data.

The common words in each category from the Chinese and English topic data are shown as word clouds in Figure 3. Background statistics are shown as a stacked bar in the center, from which we can see that the proportion distribution of each Chinese topic category was more uniform than that of English topics. The most common words within each category are shown to the left and right. The size of a word correlates with the frequency of the word used in that category. As can be seen from Figure 3, words, such as “love” and “life”, appeared frequently in all Chinese and English topics. 

Life. Chinese users posted their own photos and discussed single or weekend life, while there were many greeting posts in the English topic. The words “appearance”, “wear”, and “hair”, representing people’s physical appearance, can be seen in both Chinese and English topics, indicating the close attention to appearance in the gay community.

Entertainment. Within the entertainment topic, Chinese users focused more attention on anime, comics, and games (ACG), such as Anime Park. There were also many discussions about various horoscopes and male stars in Chinese posts. The most commonly mentioned star was Leslie Cheung, a famous bisexual singer and actor in China in the 1980s. Non-Chinese users paid more attention to photos and videos on Instagram and YouTube.

Social networking. Chinese users shared numerous stories about gay friends and experiences of meeting new gay friends. There were also many posts introducing themselves to make new friends. Non-Chinese users tended to create super groups and invite gay friends to join. There were also many posts inviting friends to follow them in the app or other social networks, such as Instagram, Facebook, and Twitter.

Relationship. High-frequency words in Chinese posts, such as “Valentine’s Day” and “Double Seventh Festival”, indicate that the Chinese users attached great importance to Valentine’s Day. The most discussed related items included the ideal boyfriend type, confessions to a boyfriend, and ending the single life. The high-frequency words in Chinese topics, such as “find” and “boyfriend”, and those in non-Chinese posts, “true” and “waiting”, reflected their desire for stable relationships.

Self-identification. Chinese users used words like “gay”, “1” (top), and “0” (bottom) to talk about their sexual identity and sexual roles. Similarly, Chinese users often used words like “not easy”, “thirty” (representing the age of a person), and “monster”, indicating that Chinese gay man have problems regarding social identity and may suffer from social discrimination. However, there were still many mutually encouraging posts represented by the word “proud.” Non-Chinese users used words like “people”, “human”, and “rights”, indicating that non-Chinese users are more active in fighting for equal rights for gay men.

Sexual behavior. Chinese users posted frequently about “AIDS” and “condom”, reflecting the effectiveness of the promotion of acquired immunodeficiency syndrome (AIDS) prevention in China. Meanwhile, Chinese users had many discussions on “hookup”, “no hookup”, and “419 (for one night)”. The words “discrimination” and “solidarity” appeared frequently in the Chinese topics while they were less common in the English topic data. Non-Chinese users talked more about HIV status, testing, and safe sex behavior.

Work and study. The word “busy” was mentioned frequently in the Chinese posts, indicating the current fast-paced lifestyle of Chinese people. In terms of study, there was considerable discussion about the college entrance examination and the graduation season. The discussion on work revolved around what they were doing and unspoken rules for the workplace. Non-Chinese users discussed recreation, taking pictures, and making money more frequently.

Marriage and family. Within the Chinese posts, the Chinese users used words like “mom”, “dad”, “thanksgiving”, “gay”, and “hope” to express their mental conflicts between fulfilling filial piety and their sexual identities. The forced marriage phenomenon was also discussed. There were fewer discussions about other brothers and sisters and children. In the non-Chinese posts, many users not only posted about their parents but also shared experiences regarding their brothers and sisters. Others shared more birthday and wedding wishes. The word “happy” was frequently used in the English posts, indicating that the non-Chinese users were generally more positive than the Chinese users.

#### 3.1.3. Sentimental and Psychological Analysis

The LIWC and Textmind score represents the ratio of the words in each category relative to the total word count of the queried text file. Table 4 shows the scores of the *Affect*, *PosEmo*, and *NegEmo* categories calculated by the LIWC and Textmind programs, in which bold letters represent relatively higher scores in each column. It is obvious that the proportion of emotional words contained in the Chinese topics was higher than that in the English topics. In the Chinese topics, sensitive topics contained a higher proportion of emotional words than non-sensitive topics, whereas this difference was not obvious in the English topics. From the perspective of positive emotions, Chinese users were more positive about relationships, while non-Chinese users were more positive in the relationship and marriage and family topics. From the perspective of negative emotions, Chinese users were negative about self-identification and sexual behaviors, while non-Chinese users were negative about relationships.

**Figure 4:**
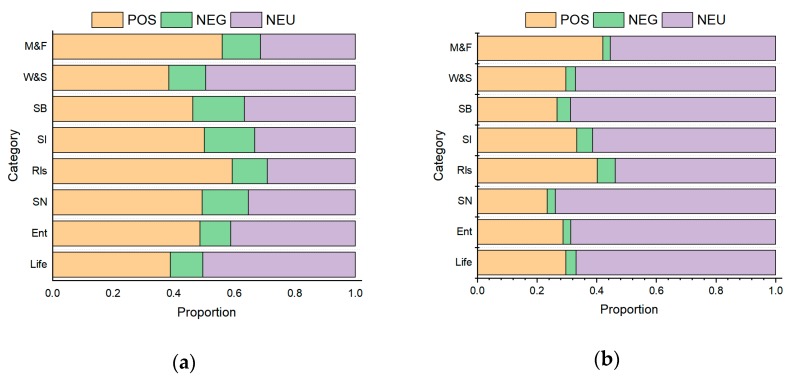
Sentiment distribution of (**a**) Chinese and (**b**) English topic data.

Figure 4 shows the sentiment distribution of the topic data. All posts in each category were classified into positive (POS), negative (NEG), and neutral (NEU). In general, positive emotions were significantly more commonly expressed than negative emotions, especially in Chinese posts. The Chinese and English topics showed the largest difference with regard to social networking while Chinese posts contained more emotions. This is because most Chinese posts were related to gay friend stories, whereas non-Chinese posts were mostly about supergroups. The proportion of affective posts in the life topic was smallest in both the Chinese and English topics, and more than 50% of the posts were neutral. The biggest differences between positive and negative emotions were found in the relationship and marriage and family topics, both in the Chinese and non-Chinese posts. The results indicate that they have positive expectations regarding their future relationships. In terms of the marriage and family topic, the positive sentiments found in the Chinese posts were related to giving thanks to parents while the positive emotions of non-Chinese posts were reflected in family celebrations.

In LIWC and Textmind, the negative emotion is divided into three categories: *Anx, Anger*, and *Sad*. Table 5 shows the scores of these negative emotions of each category calculated using the LIWC and Textmind programs, in which bold letters represent the highest score in each column. With regard to anxiety, Chinese users tend to be more anxious about self-identification while non-Chinese users were extremely anxious about sexual behaviors. In terms of anger, while it most frequently appeared in the sexual behaviors topic under Chinese posts, it commonly featured in the relationship topic under non-Chinese posts. From the perspective of sadness, the sadness of Chinese users is mainly reflected in their gay identity, since they suffered from more pressure on homosexuality in a conservative society compared to western countries. Compared to Chinese users, non-Chinese users feel sadder when dealing with relationship issues. Therefore, self-identification and sexual behaviors were main triggers of negative feelings of Chinese users, while relationships and sexual behaviors were major causes of negative feelings of non-Chinese users.

**Figure 5:**
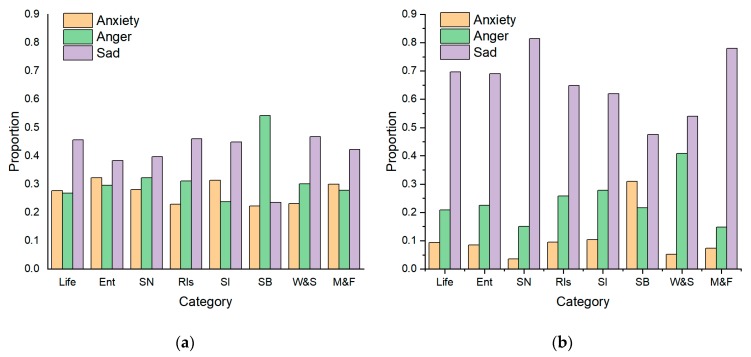
Negative emotion distribution of (**a**) Chinese and (**b**) English topic data.

Figure 5 shows the distribution of negative emotions in the topic data. Sadness was the most common negative emotion in both the Chinese and non-Chinese posts, and the Chinese posts were more negative than the non-Chinese posts. The proportional differences between the three negative emotions in the Chinese topics were smaller than in the English topics. Except for the sexual behavior topic, the Chinese users tend to be more anxious than the non-Chinese users. Similarly, except for the self-identification and work and study topics, the Chinese posts contain more anger than the non-Chinese posts. The proportion of sadness in the non-Chinese posts was significantly higher than in the Chinese posts. Moreover, the Chinese users expressed anxiety and anger more often than the non-Chinese users. In particular, the Chinese users expressed strong anger in the sexual behavior topic while the non-Chinese users expressed more sadness in this topic.

### 3.2. Voting Data Analysis

#### 3.2.1. Basic Statistical Results

Until the collection of data, a total of 114 viewpoints were published in Blued, and the statistical results of the viewpoints for the seven studied categories are presented in Table 6.

#### 3.2.2. Main Points by Category

*Life.* Socially, gay men focused more attention on the faces (54.4%) of their partners rather than on their physical shape, and 72% of the participants were willing to accept cosmetic surgery. More than 57% of the participants said they would go to the offline counter to buy skincare products, and nearly half of the participants said they could accept gays who define themselves as “tops” wearing makeup. A total of 77.5% of the participants said that they would continue to maintain their fitness after consummating a stable relationship. According to the comments on this voting item, 66.6% of the participants indicated that they work out for their own health or looks, and the workouts have nothing to do with making friends or maintaining relationships. Meanwhile, 28.8% of the participants said they believe that maintaining a good physical shape is important when participating in a relationship or making friends in a gay group. In terms of physical shape, 61.7% of the participants focused more attention on their partners’ hips than on their chests. While there were differences between gay and straight man, they did have some commonalities in their lifestyles: 79.7% of the participants indicated they like to sleep naked, 75.3% said they feel anxious when they are away from their mobile phones, and 51% were uncomfortable with people looking at an embarrassing picture of them.

Social networking. In terms of meeting new friends, 76.9% of the participants said they would respond to greetings from strangers. More than half of the participants said that they would respond to greetings even if the other person did not have an avatar. In order to protect their privacy, 64.7% of the participants said they supported using alternative accounts to make friends online. Additionally, 62% of the participants said they focused heavily on others’ appearance in daily interactions, and 73.4% thought that two people with different worldviews could not be compatible with each other. In terms of gender preference, 62% of the participants indicated that they did not mind others’ sex role, 64.4% believed that there could be a pure friendship between a “top” and a “bottom”, and 57.7% disliked being called “sister” in gay groups.

Relationship. Items related to relationships accounted for half of all voting items. We divided the voting items into dating, in a relationship, and after a breakup. In terms of dating, 64.6% of the participants said that they have no clear standard for a boyfriend, mainly basing their decisions on first appearances. A total of 67.9% of the participants said they did not oppose single people having affairs with multiple partners. When in a relationship, 62.1% of the participants said that they should show conjugal love in social networks, but 74.4% of the participants said they would not display affection in public. Further, 74.9% of the participants thought that there should be privacy between couples. More than half (53.6%) of the participants said they could not accept long-distance relationships because of the lack of safety, but in another voting item, 69.6% of the participants said they would go to where their boyfriend was located. About half (50.8%) of the participants said that they should be away from the gay group when they were in a committed relationship, and 56.8% said they could not accept an open relationship. However, 55.2% of the participants believed that they could keep “back-burner” men while they were in relationships. A total of 65.3% of the participants said that they would not trust their boyfriends to go to a gay bar alone, 61.6% said they are unable to forgive cheaters, and 67% stated that cheaters could no longer be believed. There were also many discussions about money issues in a relationship. A significant majority (73.3%) of the participants said that they ask about their potential partner’s income before committing to a relationship while more than half would mind that the other was part of “the moonlight clan” (people who expend their entire salary before the end of each month). Further, 63.5% of the participants would not put their wages together while 65% would share date expenses with their boyfriends. After a breakup, 54.7% of participants said they would not start a new relationship right away.

Self-identification. In terms of self-identification, 65.2% of the participants said that if they had another chance, they would not choose to be gay again. A total of 59.3% of the participants said that they have not kissed girls, 61.7% would not argue with each other when they encountered anti-gay speech, and 62.9% believed that homosexuals are born with their sexual preference. Effeminacy is a feature of some gay men, and 53.1% of the participants believed that gays show a feminine inclination. Moreover, 40% of the participants said they wanted to try wearing women’s clothing. In terms of sexual preference, 62% of the participants felt that one’s sexual role is unimportant, and 62.5% said they do not believe that there is a pure top in gay relationships.

Sexual behavior. A little more than half (54.3%) of the participants said that they would not interact with HIV-infected people, but more than half of the participants said that they would not give up having sex with others after they end a relationship. The large majority (88.6%) of the participants said that they would get an HIV test routinely when AIDS is curable, but only 50.52% will take regular HIV tests. Additionally, 64.2% of the participants indicated that they would reject an invitation for substance use, while 35.8% of the participants said they would like to try drugs. 

Work and study. Disclosing sexual orientation in the workplace will undoubtedly affect a gay individual’s work. Thus, 86.3% of the participants said they would hide their sexual orientation at work, although it is still possible to find gay men among their colleagues through the GSN app. A total of 56.5% of the participants said that they would meet other gay men and take care of each other, while 50.9% thought that the benefits of office love outweigh the risks. In the student group, 63.3% of the participants said they would not break up with their partners after graduation.

Marriage and family. Same-sex marriage is not currently recognized by law in China. Unsurprisingly, 68.2% of the participants said that they had not discussed homosexuality with their parents. Further, 65% of the participants said they would resist forced marriage, 53.4% said they would marry lesbians, and 72.4% said that they would break up with their boyfriend once he got married. A significant majority (78.1%) of the participants said they wanted to have a child, and 64.4% indicated that they would adopt a child. Some participants supported giving birth to a child through surrogacy, but they were afraid that their child would have psychological problems or suffer discrimination in the future. 

## 4. Discussion and Conclusions

We examined posts and voting projects on the world’s largest GSN app for gay men, Blued, to study the publicly textual content of the online gay community in depth, with a focus on investigating the daily behavioral patterns and opinions of members of the gay population. On the one hand, based on the huge number of participants in Blued—181,308,061 participants (the number of posts, comments and likes) to the topic page and 5,449,693 participants (the number of votes and comments) to the voting page—it is easy to see how popular GSN apps are in the gay community. Our analysis of the data indicated that the posts contained more positive than negative emotions. Therefore, GSN app seem to have the potential to inspire gay men to live a more active life. On the other hand, our analysis also found some potential disadvantages.

Our first research focus was theme extraction from all the posts. We divided the posts into eight categories, four of which included sensitive topics. More than 60% of the posts were related to life, especially in the English topics, followed by topics related to relationships and social networking. In fact, nearly one-fifth of the Chinese posts were related to relationships. The results reflect the social needs of users, who hope to extend the connection between each other by sharing details of their daily lives, which is very important for gay men, who cannot expose their sexual orientation on ordinary social platforms. Based on the content of the posts, it is clear that gay men are very concerned about their own appearance, body shape, and clothing. In the Chinese life topic, nearly half of the hashtags were related to selfies. When making friends, gays focus more attention on the other person’s face and body shape, whereas there is little mention of the other person’s conduct. Although face and body shape are also important in heterosexual interactions, it seems to be a greater focus in the gay community [23]. Related studies have shown that more gay men than heterosexual men feel pressure to be in good physical shape [24]. This leads to a gym culture among gay men. However, the over-emphasis of external appearance and body size within the gay community weakens the role of other factors in making friends, which may lead to unhealthy standards of spouse selection and may cause those who do not have the ideal appearance and body shape to feel inferior. In addition, relatively few posts were related to entertainment and work and study, and the content of these topics in the Chinese and non-Chinese posts varied considerably due to differences in regional cultures. 

Among the sensitive topics, less than 8% of the posts were related to gay identity, and there were even fewer such posts in the English topics. From the perspective of the theme, the Chinese users expressed self-awareness, self-encouragement, and a desire for social identity while the non-Chinese users expressed a desire for human rights and equality. Currently, same-sex marriage has been legalized in more than 20 countries; however, it is not recognized by law in China. Even posting videos related to homosexuality in mainstream media and social networks is regarded as a violation. The enormous social pressure caused by this negative attitude has led many sexual minorities to give up their self-identification in order to follow social norms. Relevant research shows that there are 16 million “tongqi” (straight woman married to gay man) in China [25]. Posts related to sexual behaviors accounted for 1.37% of the total Chinese posts, and there were even fewer in the non-Chinese posts. From the perspective of theme, most of the non-Chinese posts were related to HIV testing and safe sex, with less information related to hookups. This may be because the word “hookup” is expressed in different languages, which is difficult to understand using a translation program. Nearly 60% of the Chinese posts were related to “hookup”, and one quarter of them were asking for hookups. In fact, the number of posts asking for hookups was more than the number of posts against hooking up. A related study proved the correlation between hookups or casual sex and HIV infection [26]. However, users rarely disclose their HIV status, which further exacerbates the risk of HIV infection among gay men. At the same time, based on the high frequency of the words “AIDS” and “condom”, it may reflect the promotion of AIDS prevention in China over the past few years. However, the lack of adolescent sex education and loopholes in the age limits for minors using apps may aggravate the HIV situation.

The second research focus was on identifying emotions in each category of posts. The results suggest that most of the posts were positive. The relationship and marriage and family topics contained the most emotions, especially positive emotions. The proportion of emotional words in the Chinese posts was higher than in the non-Chinese posts in all categories. With regard to distribution patterns, the Chinese and non-Chinese posts were most similar in the life topic and most different in the social networking topic. With regard to negative emotions, both the self-identification and sexual behavior topics contained many negative emotions, but there was a greater difference in the distribution of negative emotions between the Chinese and non-Chinese posts. The results show that the Chinese posts revealed considerable anxiety in the self-identification topic, and words related to death commonly appeared in this topic. The non-Chinese posts revealed more anxiety in sexual behavior, and words related to death appeared more frequently in this topic. Other studies have shown that the incidence of mood and anxiety disorders, the main risk factors for suicidal behavior, are more strongly related to homosexuality, lesbian, or bisexuality identities rather than sexual behavior or attractiveness [27].

The third research focus was on the Chinese users’ views on sensitive issues. The voting data show that more than half of the participants would not accept open or long-distance relationships, although 55.2% supported having “back-burner” men when in a relationship. It seems that, when in a relationship, gay men are not only insecure about their partners but also fail to fully focus upon them. With regard to sexual behavior, 54.3% of participants said they would not interact with HIV-infected people, but more than half said they would not give up having sex with others after they end a relationship, and only half of the participants said they would participate in regular HIV testing. It can thus be inferred that the risk of HIV infection in this group is likely to be high. In terms of drug abuse, 64.2% of the participants said that they would reject their friends’ drug invitations. The comments on this voting item indicated that most popular drug is “Rush”, a club drug used to enhance sexual pleasure. Although “Rush” is not currently defined legally as a drug in China, its sale has recently been banned in the country. Regarding the sexual orientation of homosexuality, more than 60% of the participants said that if they had another chance, they would not want to be gay again, and more than 80% said they would hide their sexual orientation at work because they feared ridicule and discrimination. Nearly 70% of the participants said they have not discussed homosexuality with their parents. In order to maintain their reputations and avoid moral distress and social discrimination [28], more than half of the participants said they would marry a lesbian. The results show that self-identification, family acceptance, and social inclusion tend to be low for Chinese gay men.

Although GSN apps are widely used by the general population, public health practitioners and educators are just beginning to use this resource as a tool for research, education, and sharing information [29]. Our findings suggest that GSN apps can be used to reflect the behavior characteristics of gay men and identify individuals at risk of casual sex and HIV. In addition, although the social inclusion of homosexuality is presently occurring slowly in China, in the context of traditional Chinese culture, gay individuals still face tremendous discrimination and psychological pressure, especially those infected with HIV. Public information about HIV should be promoted more strongly, and sexual education should be popularized for adolescents in China.

Limitations should be considered when interpreting our findings. Our hashtag list did not cover all popular or sensitive topics. Similarly, many popular topics in minority languages were not covered in our research because of the language limitations of the researchers. In addition, we only included posts by searching hashtags in the topic interface, but private messages sent between accounts and posts without hashtags might generate different content. It is also notable that when dealing with English topics, we translated all non-English posts into English posts. As most posts were very short and contained an internet abbreviation spectrum, the machine translation results might not have been accurate and could have negatively affected our analysis. Additionally, as the gay men using the GSN apps were relatively young, the findings of this work may not represent the actual situation among more diverse gay communities.

Despite these limitations, we believe that this study supports the literature on behavior and opinion analyses of sexual minorities through a comparative analysis of themes and sentiments of posts in China and other countries, presenting the main viewpoints of gay individuals toward sensitive issues in Chinese gay communities. Our findings stress the need for continuous research in this area, especially to better understand whether and how this type of content can be used to help those who need interventions. In addition, it would be beneficial for this work to be replicated with other social apps or social networks to identify gay men in mainstream media through the topics they care about and to investigate behaviors and opinions in sexual minorities. 

## Figures and Tables

**Table 1:** Descriptions and examples of topic hashtag categories.

Category	Description	Hashtag Examples	Example of Voting Items	
			Question	Poll Options
Life	Talking about health, greetings, pets, memories, festival, photos or videos, tourism, dress up, habit, cooking, travel, plan and wish, bachelor life, etc.	#今日自拍打卡# (selfie of today) #一个人的生活# (single life)	Which do you prefer: comfort or freedom?	(1) Small city, full of happiness. (2) Big city, just be yourself.
Entertainment	Discussing male stars, movies, music, award, constellation, game, sports meeting, etc.	#型男速递# (handsome men) #奥斯卡# (Oscar)	n/a	n/a
Social networking	Searching for new friends, talking about friends, etc.	#最佳损友# (best friend) #漂流瓶# (whispering Sailing)	Do you think it’s offensive to call friends sisters?	(1) Yes. (2) No.
Relationship	Expressing preference of boyfriend, confessing to his boyfriend, trying to find a boyfriend, talking about ex-boyfriends, etc.	#我和x先生# (Mr. X and me) #最浪漫的事# (the most romantic thing)	Can you accept a long-distance relationship?	(1) Yes, true love stands the test of distance. (2) No, lack of safety.
Self-identification	Expressing a sense of pride in their identities, talking about sexual orientation, coming out, social support, etc.	#爱自己# (love yourself) #同志骄傲日# (Gay Pride day)	Will you argue with each other when you encounter anti-gay speech?	(1) Yes. (2) No.
Sexual behavior	Talking about HIV detection and precaution measures, drug abuse, etc.	#为艾发声# (speak for AIDS) #青春零艾滋# (no AIDS in youth)	Will you interact with HIV-infected people?	(1) Yes, he can live normally if he takes medicine regularly. (2) No, I don’t want to risk myself no matter how I like him.
Work & studies	Sharing experience in school, university or workplace, etc.	#我的校园生活# (my school life) #职场潜规则# (hidden rules in workplace)	Will you hide your sexual orientation at work?	(1) Yes, for protecting my privacy. (2) No, just be myself.
Marriage & family	Expressing emotion about parents, brothers and sisters, sharing opinions about marriage and child, etc.	#父母老了# (parents getting old) #我们结婚吧# (let’s get married)	Will you adopt a child in the future?	(1) Yes. (2) No.

**Table 2:** Top 20 languages analyzed in the posts data.

Language Name	Native Name	Number of Posts	Language Name	Native Name	Number of Posts
Chinese	中文	935,745	German	Deutsch	8807
English	English	482,110	Malay	Bahasa, Melayu, بهاس ملايو	8337
Portuguese	Português	65,107	Dutch, Flemish	Nederlands, Vlaams	4117
Spanish, Castilian	Español	29,775	Galician	Galego	3148
Vietnamese	Tiếng Việt	27,943	Latin	latine, lingua latina	3085
Thai	ไทย	27,904	Javanese	ꦧꦱꦗꦮ, Basa Jawa	2949
Indonesian	Bahasa Indonesia	24,280	Polish	język polski, polszczyzna	2926
Tagalog	Wikang Tagalog	13,073	Japanese	日本語 (にほんご)	2618
Italian	Italiano	10,263	Finnish	suomi, suomen kieli	2599
French	français	9720	Danish	dansk	2201

**Table 3:** The statistical results of topic categories.

Category	Number of Topics		Posts Released		Users Involved	
	CHN	ENG	CHN	ENG	CHN	ENG
Life (Life)	272	76	567,062(60.9%)	398,480(74.9%)	402,257	31,009
Entertainment (Ent)	50	6	55,998(6.0%)	17,697(3.3%)	38,853	7245
Social networking (SN)	15	8	28,102(3.0%)	39,350(7.4%)	20,478	10,130
Relationship (Rls)	107	6	178,461(19.2%)	38,144(7.2%)	143,629	11,736
Self-identification(SI)	41	2	71,743(7.7%)	10,652(2.0%)	61,719	4559
Sexual behavior (SB)	9	1	12,799(1.4%)	2822(0.5%)	9280	1574
Work and study (W and S)	8	3	9833(1.1%)	12,506(2.4%)	8048	5643
Marriage and family (M and F)	9	2	7111(0.8%)	12,530(2.4%)	6345	5109
Summary	511	104	931,109	532,181	690,609	77,005

**Table 4:** Affection scores of each category calculated by LIWC programs.

Category	Affection (%)		Positive Emotion (%)		Negative Emotion (%)	
	CHI	ENG	CHI	ENG	CHI	ENG
Life	5.8	1.9	3.4	1.6	1.3	0.2
Entertainment	6.3	1.6	3.8	1.4	1.4	0.1
Social networking	5.9	1.7	3.1	1.5	1.4	0.2
Relationship	**7.9**	**2.6**	**4.7**	**2.2**	1.6	**0.4**
Self-identification	**7.4**	2.1	4.0	1.7	**1.8**	0.3
Sexual behavior	**8.0**	1.7	3.9	1.4	**1.8**	0.3
Work and study	5.2	1.8	3.0	1.5	1.3	0.2
Marriage and family	**7.8**	2.5	4.0	**2.3**	1.5	0.2

**Table 5:** Scores of negative emotions of each category calculated by the LIWC programs.

Category	Anxiety (‰)		Anger (‰)		Sadness (‰)	
	CHI	ENG	CHI	ENG	CHI	ENG
Life	2.1	0.2	2.0	0.3	3.8	1.1
Entertainment	2.3	0.1	2.3	0.2	3.2	0.7
Social networking	2.3	0.1	2.6	0.2	3.4	1.2
Relationship	2.1	0.3	2.8	**0.8**	4.7	**2.1**
Self-identification	**3.6**	0.2	2.9	0.6	**5.9**	1.5
Sexual behavior	2.3	**0.7**	**6.6**	0.5	3.0	1.7
Work and study	1.8	0.1	2.4	0.5	3.9	0.7
Marriage and family	2.7	0.1	2.4	0.2	3.9	1.2

**Table 6:** The statistical results of the voting categories.

Category	Number of Viewpoints	Number of Votes	Number of Comments
Life	18(15.8%)	576,839	49,615
Social networking	15(13.2%)	726,010	52,143
Relationship	57(50.0%)	2,529,860	204,600
Self-identification	7(6.1%)	405,017	36,819
Sexual behavior	5(4.4%)	198,168	17,040
Work & study	5(4.4%)	261,642	20,205
Marriage & family	7(6.1%)	344,704	27,031
Summary	114	5,042,240	407,453

## Appendix A

**Table A1:** A comprehensive list of voting questionnaires on Blued.

ID	Question	Option 1	Option 2
**Life**			
1	Which do you prefer: comfort or freedom?	Small city, full of happiness.	Big city, just be yourself.
2	Will you continue to work out after consummating a stable relationship?	Yes, I will be attractive if I look handsome.	No, I will indulge myself.
3	Can you accept 1 wearing makeup?	Yes.	No.
4	Can you accept massages by strangers in bathhouse?	Yes, it’s good social manners.	No, it’s embarrassing.
5	Which would you choose: artificial beauty and natural ugliness?	Artificial beauty.	Natural ugliness.
6	Which one do you like: nice ass or muscular chests?	Yes.	No.
7	Have you ever had a wet dream staying with a male star?	Yes.	No.
8	Which do you value more: body shape or face value?	Body shape.	Face value.
9	Do you like to sleep naked?	Yes.	No.
10	Have you ever thought about wearing women’s clothing?	Yes.	No.
11	Are you suffering from mobile phone separation anxiety?	Yes.	No.
12	Do you mind if others see your previous embarrassing pictures?	Yes.	No.
13	Will you spend ahead of time to celebrate the upcoming Chinese New Year?	Yes.	No.
14	Will you give the elders a red envelope in Chinese New Year after having job?	Yes.	No.
15	Will you go to the offline counter to buy skincare products?	Yes, just be myself.	No, that’s embarrassing.
16	Will you take photos with friends who looks better than yourself?	Yes, I will smile.	No, I will refuse.
17	Is it your style to be different?	Yes.	No.
18	Which do you prefer: masturbation cup or vibrator?	Masturbation cup.	Vibrator.
**Making Friends**			
19	Is the sexual role 1 (top) and 0 (bottom) important when making friends?	Yes, I will blacklist him if we are in the same sexual role.	No, I just want to meet someone interesting.
20	Do you care about the other person’s face value?	Yes, friends should be handsome.	No, true heart is more important.
21	Will you respond to greetings if the other person did not have an avatar?	Yes.	No.
22	Should I tell my fiend if his boyfriend cheated on him?	Yes, it’s our duty.	No, I don’t want to bother myself about others.
23	If your idol become a WeChat salesmen, will you still love him?	Yes.	No.
24	Will you respond to strangers who greeting like "Are you online"?	Yes, I will response politely.	No, I just want to talk to those who is sincere.
25	Will you tell your friend if he bought a fake item?	Yes, I will kindly remind him to pay attention to being deceived.	No, it’s embarrassing.
26	Will you accept friend requests from the person who unfriend you?	Yes.	No.
27	Do you think it’s offensive to call friends sisters?	Yes.	No.
28	Do you think two people with different worldviews can be compatible with each other?	Yes.	No.
29	When making friends, will you consider where they come from?	Yes.	No.
30	Is there a pure friendship between 1 and 0?	Yes.	No.
31	Will you choose a private place when you meet gay friend for the first time?	Yes.	No.
32	Is that all right to use alternative accounts to make friends online?	Yes.	No.
33	Can you accept the lubricant is not effective?	Yes.	No.
**Relationship**			
34	Can you stay friends after a breakup?	Yes, friendship remains even if we break up.	No, I’ll blacklist him.
35	Should single men blame themselves on having affairs with multiple partners?	Yes, I can’t stand that.	No, it’s none of your business.
36	“Tiancai” boyfriend (a man who is totally your type) is a playboy, can you accept?	Yes.	No.
37	Can you forgive your boyfriend if he cheated on you?	Yes, but don’t do it next time.	No, old habits die hard.
38	Can you accept a long-distance relationship?	Yes, true love stands the test of distance.	No, lack of safety.
39	Should you check your boyfriends’ phone?	Yes, secret is a time bomb.	No, there should be free space between couples.
40	Do you trust your boyfriend to go to Gay bar alone?	Yes, he is not dare.	No, I know he can’t control himself.
41	Do you accept sharing date expenses with your boyfriends?	Yes, I support separate finances.	No.
42	Will you confess to the men who you have secret crushes on?	Yes, don’t leave any regrets behind.	No, I’ll keep him in my heart.
43	Are straight men an aphrodisiac or poison to you?	Aphrodisiac.	Poison.
44	Do you mind if your boyfriend share photos of his body shape on the Internet?	Yes.	No.
45	Do you want to get away from gay communities after going steady with someone?	Yes, for safety.	No, true love stands the test of interference.
46	Should gay men show conjugal love in social networks?	Yes, love should be seen by someone.	No.
47	Do you mind if your boyfriend is in contact with ex-boyfriend?	Yes.	No.
48	Do you mind if your boyfriend is “the moonlight clan” (people who expend their entire salary before the end of each month)?	Yes.	No.
49	After the quarrel, should 1 compromise first?	Yes.	No.
50	Will you pay attention to the status of your ex-boyfriend?	Yes.	No.
51	Will you get mad when your boyfriend pays more attention to their phone than you?	Yes.	No.
52	Do you accept open relationships?	Yes.	No.
53	Should there be any "back-burner" men while you are in a relationship?	Yes, to avoid risk.	No, one life, one love.
54	If a man you like is boyfriend of your good friend, will you chase him?	Yes, just be myself.	No, bless for my friend.
55	If you find your boyfriend and you have a common friend in gay group, will you gossip about it?	Yes, I’m curious about sensitive relationship.	No, I don’t care.
56	Do you want to start a new relationship quickly after a breakup?	Yes.	No, I need time to calm down.
57	Will you lower your standard for a boyfriend if you are single for a long time?	Yes, every man has his fault.	No, there will always be someone worth waiting for.
58	Should I respond to my ex-boyfriend who want to get back together?	Yes.	No.
59	Will you share a house with people who like bring people home?	Yes.	No.
60	Will you contact your first love if he becomes less handsome after grew up?	Yes.	No.
61	How to do if your boyfriend shows bad sexual skills at date night?	I need to be calm, I am desperate.	It’s not a big deal, I will help you with my passion.
62	Will you confess to your boyfriend the history of your love?	Yes.	No.
63	Will you go out with your boyfriend if he looks not handsome?	Yes.	No.
64	Do you have virginity obsession?	Yes.	No.
65	Will you display affection in public?	Yes.	No.
66	Can you accept platonic love?	Yes, ideal love is what I pursue.	No, we are human.
67	Do you dare to test your boyfriend’s loyalty?	Yes.	No.
68	Will you masturbate without your boyfriend knowing?	Yes.	No.
69	Have you ever been confessed by girl?	Yes.	No.
70	Do you like to warm up before having sex?	Yes.	No.
71	Is male masculinism good or bad?	Good.	Bad.
72	Have you ever kissed a girl?	Yes.	No.
73	Should you return valuable gifts to your partner after a breakup?	Yes.	No.
74	Will you go to work where your boyfriend located?	Yes.	No.
75	Will you ask about your partner’s income before committing to a relationship?	Yes.	No.
76	Are you obsessed with sports boys?	Yes.	No.
77	Will you break up because of disharmony in sexual intercourse?	Yes.	No.
78	Should there be privacy between couples?	Yes.	No.
79	Should older virgins be self-confident or inferior?	Self-confident.	Inferior.
80	Which do you prefer: men from the South or men from the North?	The men in the South.	The men in the North.
81	Should people be thankful spending money on his boyfriend?	Yes.	No.
82	Will you put your boyfriends’ and your wages together after going steady?	Yes.	No.
83	Can you believe those who cheated before?	Yes.	No.
84	Do you think it’s necessary to divide household chores clearly in husband & husband life?	Yes.	No.
85	Would you choose to confess on Valentine’s Day?	Yes.	No.
86	Is it important to have a clear standard when looking for a boyfriend?	Yes, standard is important.	No, mainly basing on first appearances.
87	Will you love someone because he is good to you?	Yes.	No.
88	Will you share your passport number with your boyfriend if he asks?	Yes.	No.
89	Will you have sex with someone when you have prostatitis?	Yes.	No.
90	Will you blame your boyfriend’s inability during of intercourse because of tiredness of journey?	Yes.	No.
**Self-Identification**			
91	Do you believe in pure 1?	Yes, there should be pure 1.	No, pure 1 is just a legend.
92	If you have another chance, will you be a gay again?	Yes, I’m not regret.	No, I just want simple life.
93	Can you accept that 0 is taller than 1?	Yes.	No.
94	Do you believe that straight men can be turned into gay men?	Yes.	No.
95	Will you argue with each other when you encounter anti-gay speech?	Yes.	No.
96	Do you agree that all gays show feminine inclinations?	Yes.	No.
97	Do you think homosexuals are born with their sexual preference?	Yes.	No.
**Sexual Behavior**			
98	Will you interact with HIV-infected people?	Yes, he can live normally if he takes medicine regularly.	No, I don’t want to risk myself no matter how I like him.
99	Will you give up having sex with others after end a relationship?	Yes, I’ll wait for Mr. right.	No.
100	Do you take regular HIV tests?	Yes.	No.
101	will you take HIV tests routinely when AIDS is curable?	Yes.	No.
102	Will you reject their friends’ drug invitations?	Yes.	No.
**Work & Study**			
103	Will you recognize each other when you meet gay friends in your work?	Yes, take care with each other.	No, just do my own part.
104	Will you hide your sexual orientation at work?	Yes, for protecting my privacy.	No, just be my self.
105	Will you share a bed with a straight male colleague?	Yes.	No.
106	Is office love good or bad?	Good.	Bad.
107	Will you break up with your boyfriend because of graduating from university?	Yes.	No.
**Marriage & Family**			
108	If you your encounter a relative in Blued, will you blacklist him?	Yes, it’s embarrassing.	No, we’ll take care of each other.
109	As a gay, do you want to have a child?	Yes, I want to be a father.	No, just two of us.
110	If your boyfriend gets married with another person, will you break up with him?	Yes, I will break up with him.	No, true heart is more important.
111	Have you ever discussed homosexuality with your parents?	Yes, proper guidance makes parents more open-minded.	No, that’s a sensitive topic.
112	Will you adopt a child in the future?	Yes.	No.
113	Will you choose to marry a lesbian?	Yes.	No.
114	In terms of forced marriage, will you obedient or rebellious?	Obedient.	Rebellious.
